# Some majorization integral inequalities for functions defined on rectangles

**DOI:** 10.1186/s13660-018-1739-2

**Published:** 2018-06-27

**Authors:** Shanhe Wu, Muhammad Adil Khan, Abdul Basir, Reza Saadati

**Affiliations:** 1 Department of Mathematics, Longyan University, Longyan, China; 2 Department of Mathematics, University of Peshawar, Peshawar, Pakistan; 3 Department of Mathematics, Iran University of Science and Technology, Tehran, Iran

**Keywords:** 26A51, 26D15, 26D20, Convex function, Coordinate convex function, Rectangle, Majorization, Favard’s inequality

## Abstract

In this paper, we first prove an integral majorization theorem related to integral inequalities for functions defined on rectangles. We then apply the result to establish some new integral inequalities for functions defined on rectangles. The results obtained are generalizations of weighted Favard’s inequality, which also provide a generalization of the results given by Maligranda et al. (J. Math. Anal. Appl. 190:248–262, 1995) in an earlier paper.

## Introduction

There is a certain intuitive appeal to the vague notion that the components of an *n*-tuple **x** are less spread out, or more nearly equal, than the components of an *n*-tuple **y**. The notion arises in a variety of contexts, and it can be made precise in a number of ways. In remarkably many cases, the appropriate statement is that **x** is majorized by **y** (or **y** majorizes **x**). Namely, for two *n*-tuples $\mathbf{x}=(x_{1},x_{2}, \dots ,x_{n})$ and $\mathbf{y}=(y_{1},y_{2},\dots ,y_{n})$, **x** is said to be majorized by **y** (denoted $\textbf{x} \prec \textbf{y}$) if $\sum_{i=1}^{m}x_{[i]}\leq \sum_{i=1} ^{m}y_{[i]}$ for $m=1,2,\dots ,n-1$ and $\sum_{i=1}^{n}x_{i}=\sum_{i=1} ^{n}y_{i}$, where $x_{[1]}\geq x_{[2]}\geq \cdots \geq x_{[n]}$ and $y_{[1]}\geq y_{[2]}\geq \cdots \geq y_{[n]}$ are rearrangements of **x** and **y** in descending order. A mathematical origin of majorization is illustrated by the work of Schur [35] on Hadamard’s determinant inequality. Many mathematical characterization problems are known to have solutions that involve majorization. Complete and superb references on the subject are the books [9, 28]. The comprehensive survey by Ando [7] provides alternative derivations, generalizations, and a different viewpoint.

The following theorem is known in the literature as the majorization theorem (see [20, 22, 23, 33, 35]).

### **Theorem** 1.1

*Let* $f:I\rightarrow \mathbb{R}$ *be a continuous convex function on the interval* *I*, *and let* $\mathbf{x}=(x_{1},x_{2},\dots ,x_{n})$ *and* $\mathbf{y}=(y_{1},y_{2},\dots ,y_{n})$ *be two n*-*tuples such that* $x_{i},y_{i}\in I$ ($i=1,2,\dots ,n$). *If* **x** *is majorized by* **y**, *then*

1.1 $$ \sum_{i=1}^{n} f ( x_{i} ) \leq \sum_{i=1}^{n} f ( y_{i} ) . $$

The inequality asserted by Theorem 1.1 is also called majorization inequality. It is an inequality in elementary algebra for convex real-valued functions defined on an interval of the real line, and it generalizes the finite form of Jensen’s inequality. This majorization ordering is equivalently described in Kemperman’s review [25]. An extension of this fact for arbitrary real weights and decreasing *n*-tuples **x** and **y** can be found in [19]. General results of this type are obtained by Dragomir [17] and Niezgoda [30]. In recent years, many formulas such as Taylor formula, Hermite interpolating, Montgommery identities and inequalities for means, etc. have been used and generalized by majorization inequalities for *n*-convex functions; see [1–8, 10–15, 21, 24, 29, 31, 36, 37, 41–45] and references therein.

Recently, it has come to our attention that certain integral majorization theorems, we begin with recalling some relevant results. In 1947, Fuchs [19] gave the following integral majorization theorem for convex functions and two monotonic sequences.

### **Theorem** 1.2

([19])

*Let* $\kappa (\tau ), \nu (\tau ) : [a, b]\rightarrow \mathbb{R}$ *be continuous and increasing functions*, *and let* $\mu :[a, b]\rightarrow \mathbb{R}$ *be a function of bounded variation*.

(i) *If*
  $$ \int_{x}^{b} \kappa (\tau )\,d\mu (\tau ) \leq \int_{x}^{b} \nu (\tau )\,d\mu (\tau )\quad \textit{for }x\in [a, b] $$
  *and*
  $$\begin{aligned} \int_{a}^{b} \kappa (\tau )\,d\mu (\tau ) = \int_{a}^{b} \nu ( \tau )\,d\mu (\tau ), \end{aligned}$$
  *then for every continuous convex function* *ϕ*, *we have*
  1.2 $$ \int_{a}^{b} \phi \bigl[ \kappa (\tau ) \bigr]\,d\mu (\tau )\leq \int_{a}^{b} \phi \bigl[ \nu (\tau ) \bigr]\,d\mu ( \tau ). $$
(ii) *If*
  $$ \int_{x}^{b} \kappa (\tau )\,d\mu (\tau ) \leq \int_{x}^{b} \nu (\tau )\,d\mu (\tau ) \quad \textit{for }x\in [a, b], $$
  *then for every continuous increasing convex function* *ϕ*, *we have*
  1.3 $$ \int_{a}^{b} \phi \bigl[ \kappa (\tau ) \bigr]\,d\mu (\tau ) \leq \int_{a}^{b} \phi \bigl[ \nu (\tau ) \bigr]\,d\mu ( \tau ). $$

In 1995, Maligranda, Pečarić, and Persson [27] established the following analogue of the Fuchs inequality.

### **Theorem** 1.3

([27])

*Let* *w* *be a weight function*, *and let* *f* *and* *g* *be positive integrable functions on* $[a, b]$. *Suppose that* $\varphi : [0, \infty )\rightarrow \mathbb{R}$ *is a convex function and that*

$$ \int_{a}^{x}f(t) w(t)\,dt \leq \int_{a}^{x}g(t) w(t)\,dt\quad \textit{for }x\in [a, b] $$

*and*

$$ \int_{a}^{b}f(t) w(t)\,dt = \int_{a}^{b}g(t) w(t)\,dt. $$

(i) *If* *f* *is a decreasing function on* $[a, b]$, *then*
  1.4 $$ \int_{a}^{b}\varphi \bigl[ f(t) \bigr] w(t)\,dt \leq \int _{a}^{b}\varphi \bigl[ g(t) \bigr] w(t)\,dt. $$
(ii) *If* *g* *is an increasing function on* $[a, b]$, *then*
  1.5 $$ \int_{a}^{b}\varphi \bigl[ g(t) \bigr] w(t)\,dt \leq \int _{a}^{b}\varphi \bigl[ f(t) \bigr] w(t)\,dt. $$

In 1933, Favard [18] proved the following results.

### **Theorem** 1.4

*Let* Φ *be a nonnegative continuous concave function on* $[a,b]$, *not identically zero*, *and let* ϒ *be a convex function on* $[0,\widetilde{\Phi } ]$, *where*

$$ \widetilde{\Phi } = \frac{2}{b-a} \int_{a}^{b}\Phi (x)\,dx. $$

*Then*

1.6 $$ \frac{1}{b-a} \int_{a}^{b}\Upsilon \bigl[ \Phi (x) \bigr]\,dx \leq \int_{0}^{1}\Upsilon ( s\widetilde{\Phi } )\,ds. $$

As a consequence of Theorem 1.4, the following inequality was also established in [18].

### **Theorem** 1.5

*Let* Φ *be a concave nonnegative function on* $[a,b]\subset \mathbb{R}$. *If* $q>1$, *then*

1.7 $$ \frac{1}{b-a} \int_{a}^{b} \Phi^{q}(x)\,dx \leq \frac{2^{q}}{q+1} \biggl( \frac{1}{b-a} \int_{a}^{b}\Phi (x)\,dx \biggr) ^{q}. $$

Maligranda, Pečarić, and Persson [27] gave the following generalization of the Favard inequality.

### **Theorem** 1.6

([27])

(i) *Let* Φ *be a positive increasing concave function on* $[a, b]$, *and let* ϒ *be a convex function on* $[0, \widetilde{\Phi }]$, *where*
  $$ \widetilde{\Phi }=(b-a) \int_{a}^{b} \Phi (t)w(t)\,dt \Big/ \int_{a}^{b} (t-a)w(t)\,dt. $$
  *Then*
  1.8 $$ \frac{1}{b-a} \int_{a}^{b} \Upsilon \bigl[\Phi (t)\bigr]w(t)\,dt\leq \int _{0}^{1}\Upsilon (s\widetilde{\Phi })w \bigl[a(1-s)+bs\bigr]\,ds. $$
(ii) *Let* Φ *be a positive decreasing concave function on* $[a, b]$, *and let* ϒ *be a convex function on* $[0, \widetilde{\Phi }]$, *where*
  $$ \widetilde{\Phi }=(b-a) \int_{a}^{b} \Phi (t)w(t)\,dt \Big/\int_{a}^{b} (b-t)w(t)\,dt. $$
  *Then*
  1.9 $$ \frac{1}{b-a} \int_{a}^{b} \Upsilon \bigl[\Phi (t)\bigr]w(t)\,dt\leq \int _{0}^{1}\Upsilon (s\widetilde{\Phi })w \bigl[as+b(1-s)\bigr]\,ds. $$

For further results related to generalizations, extensions, and refinements of the integral inequalities of majorization type, we refer the reader to [1–6, 26, 28, 32, 34, 38–40, 46–48].

In this paper, we extend majorization and Favard inequalities from functions defined on intervals to functions defined on rectangles. The results presented in this paper generalize the results of Maligranda, Pečarić, and Persson [27].

## Preliminaries

In this section, we introduce some notions and lemmas.

### Definition 2.1

*A function* $\phi :\Omega \rightarrow \mathbb{R}$ *defined on a convex subset* Ω *of* $\mathbb{R}^{n}$ *is said to be convex if*

2.1 $$ \phi \bigl(\lambda \mathbf{x}+(1-\lambda )\mathbf{y} \bigr)\leq \lambda \phi (\mathbf{x})+(1-\lambda )\phi (\mathbf{y}) $$

*for all* $\mathbf{x}, \mathbf{y}\in \Omega $ *and* $\lambda \in [0,1]$.

In this paper, convex functions considered are supposed to be twice differentiable. It is well know that if the function *ϕ* is convex, then

2.2 $$ \phi (\mathbf{x})-\phi (\mathbf{y})\geq \nabla_{+} \phi ( \mathbf{y}) (\mathbf{x}-\mathbf{y})\quad \mbox{for all }\mathbf{x},\mathbf{y}\in \Omega , $$

where

$$ \nabla_{+}\phi (\mathbf{y}) (\mathbf{x}-\mathbf{y})=\biggl\langle \frac{ \partial \phi_{+}(\mathbf{y})}{\partial \mathbf{y}},(\mathbf{x}- \mathbf{y})\biggr\rangle ,\qquad \frac{\partial \phi_{+}(\mathbf{y})}{\partial \mathbf{y}}= \biggl(\frac{\partial \phi_{+}(\mathbf{y})}{\partial y_{1}},\frac{ \partial \phi_{+}(\mathbf{y})}{\partial y_{2}},\ldots,\frac{\partial \phi_{+}(\mathbf{y})}{\partial y_{n}}\biggr), $$

$\mathbf{x}=(x_{1},x_{2},\ldots,x_{n})$, $\mathbf{y}=(y_{1},y_{2},\ldots,y _{n})\in \Omega $, and $\langle \cdot ,\cdot \rangle $ is the usual inner product in $\mathbb{R}^{n}$.

In the literature, there are many generalizations of convex functions in different directions. One of them is coordinate convex functions introduced by Dragomir [16].

### Definition 2.2

([16])

*Let us consider a bidimensional interval* $S=[a,b]\times [c,d]\subset \mathbb{R}^{2}$. *A function* $\phi :S\rightarrow \mathbb{R}$ *is said to be coordinate convex if the partial functions* $\phi_{y}:[a,b]\rightarrow \mathbb{R}$ *defined as* $\phi_{y}(u)=\phi (u,y)$ *and* $\phi_{x}:[c,d] \rightarrow \mathbb{R}$ *defined as* $\phi_{x}(v)=\phi (x,v)$ *are convex where defined for all* $y\in [c,d]$ *and* $x\in [a,b]$.

### Lemma 2.3

([16])

*Every convex function* $\phi :S\rightarrow \mathbb{R}$ *is coordinate convex*.

### Lemma 2.4

([27])

*Let* *v* *be a weight function on* $[a,b]$.

(i) *If* *h* *is a decreasing function on* $[a,b]$, *then*
  2.3 $$ \int_{a}^{b}h(t)v(t)\,dt \int_{a}^{x}v(t)\,dt\leq \int_{a}^{x}h(t)v(t)\,dt \int_{a}^{b}v(t)\,dt \quad \textit{for }x\in [a,b]. $$
(ii) *If* *h* *is an increasing function on* $[a,b]$, *then*
  2.4 $$ \int_{a}^{x}h(t)v(t)\,dt \int_{a}^{b}v(t)\,dt\leq \int_{a}^{b}h(t)v(t)\,dt \int_{a}^{x}v(t)\,dt \quad\textit{for }x\in [a,b]. $$

## Majorization inequalities for functions defined on rectangles

In this section, we establish some majorization integral inequalities for functions defined on rectangles. The following theorem is a generalization of Theorem 1.3 mentioned in the Introduction.

### **Theorem** 3.1

*Let* *w* *and* *p* *be positive continuous functions on* $[a,b]$ *and* $[c,d]$ *respectively*, *and let* *f*, *g* *and* *h*, *k* *be positive differentiable functions on* $[a,b]$ *and* $[c,d]$ *respectively*. *Suppose that* $\phi :[0,\infty )\times [0,\infty ) \rightarrow \mathbb{R}$ *is a convex function and that*

$$\begin{aligned} \int_{a}^{x}g(t)w(t)\,dt\leq \int_{a}^{x}f(t)w(t)\,dt\quad \textit{for }x\in [a,b], \end{aligned}$$

$$\begin{aligned} \int_{c}^{y}k(s)p(s)\,ds\leq \int_{c}^{y}h(s)p(s)\,ds \quad \textit{for }y\in [c,d], \end{aligned}$$

$$\begin{aligned} \int_{a}^{b}g(t)w(t)\,dt= \int_{a}^{b}f(t)w(t)\,dt, \end{aligned}$$

*and*

$$\begin{aligned} \int_{c}^{d}k(s)p(s)\,ds= \int_{c}^{d}h(s)p(s)\,ds. \end{aligned}$$

(i) *If* *g* *and* *k* *are decreasing functions on* $[a,b]$ *and* $[c,d]$ *respectively*, *then*
  3.1 $$ \int_{a}^{b} \int_{c}^{d}\phi \bigl(g(t),k(s) \bigr)w(t)p(s)\,dt\,ds \leq \int_{a}^{b} \int_{c}^{d}{\phi \bigl(f(t),h(s) \bigr)w(t)p(s)\,dt\,ds}. $$
(ii) *If* *f* *and* *h* *are increasing functions on* $[a,b]$ *and* $[c,d]$ *respectively*, *then*
  3.2 $$ \int_{a}^{b} \int_{c}^{d}\phi \bigl(f(t),h(s) \bigr)w(t)p(s)\,dt\,ds \leq \int_{a}^{b} \int_{c}^{d}{\phi \bigl(g(t),k(s) \bigr)w(t)p(s)\,dt\,ds}. $$

### Proof

(i) Since $\phi :[0,\infty )\times [0,\infty )\rightarrow \mathbb{R}$ is a convex function, we have
  $$\begin{aligned} &\phi (x,y)-\phi (w,z)\geq \bigl\langle \nabla_{+}\phi (w,z),(x-w,y-z) \bigr\rangle ,\quad \forall (x,y),(w,z)\in [0,\infty )\times [0, \infty ) \\ &\quad \Longleftrightarrow\quad \phi (x,y)-\phi (w,z)\geq \frac{\partial \phi_{+}(w,z)}{\partial w}(x-w)+\frac{ \partial \phi_{+}(w,z)}{\partial z}(y-z). \end{aligned}$$
  Put $x= f(t)$, $y= h(s)$, $w= g(t)$, $z=k(s)$ in the last inequality and assume that
  $$\begin{aligned} \psi^{1}_{s}(t)=\frac{\partial \phi (u,v)}{\partial u}\bigg|_{u=g(t), v=k(s)},\qquad \psi^{3}_{s}(t)=\frac{\partial^{2}\phi (u,v)}{\partial^{2} u} \bigg|_{u=g(t), v=k(s)}, \\ \psi^{2}_{s}(t)=\frac{\partial \phi (u,v)}{\partial v} \bigg|_{u=g(t), v=k(s)},\qquad \psi^{4}_{s}(t)=\frac{\partial^{2}\phi (u,v)}{ \partial^{2} v} \bigg|_{u=g(t),v=k(s)}. \end{aligned}$$
  Then we have
  3.3 $$ \phi \bigl(f(t),h(s) \bigr)-\phi \bigl(g(t),k(s) \bigr) \geq \psi ^{1}_{s}(t) \bigl(f(t)-g(t)\bigr)+ \psi^{2}_{s}(t) \bigl(h(s)-k(s)\bigr). $$
  Set
  $$ F(x)= \int_{a}^{x}\bigl(f(t)-g(t)\bigr)w(t)\,dt,\quad x\in [a,b] $$
  and
  $$ G(y)= \int_{c}^{y}\bigl(h(s)-k(s)\bigr)p(s)\,ds, \quad y\in [c,d]. $$
  Then, from the assumptions in Theorem 3.1 we conclude that
  $$\begin{aligned} &F(x)\geq 0,\qquad G(y)\geq 0\quad \mbox{for }x\in [a,b], y\in [c,d], \\ &F(a)=F(b)=G(c)=G(d)= 0. \end{aligned}$$
  Multiplying both sides of inequality (3.3) by $w(t)p(s)$, we get
  3.4 $$\begin{aligned} &\bigl[\phi \bigl(f(t),h(s)\bigr)-\phi \bigl(g(t),k(s)\bigr) \bigr]w(t)p(s) \\ &\quad \geq \psi^{1}_{s}(t)\bigl[f(t)-g(t) \bigr]w(t)p(s)+\psi^{2}_{s}(t)\bigl[h(s)-k(s)\bigr]w(t)p(s). \end{aligned}$$
  Integrating both sides of inequality (3.4) gives
  $$\begin{aligned} & \int_{a}^{b} \int_{c}^{d} \bigl[\phi \bigl(f(t),h(s)\bigr)-\phi \bigl(g(t),k(s)\bigr) \bigr]w(t)p(s)\,dt\,ds \\ &\quad \geq \int_{a}^{b} \int_{c}^{d}\psi^{1}_{s}(t) \bigl[f(t)-g(t)\bigr] w(t)p(s)\,dt\,ds \\ &\quad \quad {}+ \int_{a}^{b} \int_{c}^{d}\psi^{2}_{s}(t) \bigl[h(s)-k(s)\bigr] w(t)p(s)\,dt\,ds. \end{aligned}$$
  By Fubini’s theorem we have
  $$\begin{aligned} &\int_{a}^{b} \int_{c}^{d} \bigl[\phi \bigl(f(t),h(s)\bigr)-\phi \bigl(g(t),k(s)\bigr) \bigr]w(t)p(s)\,dt\,ds \\ &\quad \geq \int_{c}^{d}p(s) \biggl[ \int_{a}^{b}\psi^{1}_{s}(t)\,dF(t) \biggr]\,ds+ \int _{a}^{b}w(t) \biggl[ \int_{c}^{d}\psi^{2}_{s}(t)\,dG(s) \biggr]\,dt. \end{aligned}$$
  Using integration by parts, we obtain
  $$\begin{aligned} &\int_{a}^{b} \int_{c}^{d} \bigl[\phi \bigl(f(t),h(s)\bigr)-\phi \bigl(g(t),k(s)\bigr) \bigr]w(t)p(s)\,dt\,ds \\ &\quad \geq \int_{c}^{d}p(s) \biggl[\psi^{1}_{s}(t)F(t) \bigg|_{a}^{b}- \int_{a} ^{b}\psi^{3}_{s}(t)g'(t)F(t)\,dt \biggr]\,ds \\ &\quad \quad {}+ \int_{a}^{b}w(t) \biggl[\psi^{2}_{s}(t)G(s) \bigg|_{c}^{d}- \int_{c}^{d} \psi^{4}_{s}(t)k'(s)G(s)\,ds \biggr]\,dt, \end{aligned}$$
  which yields
  3.5 $$\begin{aligned} &\int_{a}^{b} \int_{c}^{d} \bigl[\phi \bigl(f(t),h(s)\bigr)-\phi \bigl(g(t),k(s)\bigr) \bigr]w(t)p(s)\,dt\,ds \\ &\quad \geq - \int_{c}^{d} \int_{a}^{b}\psi^{3}_{s}(t) g'(t)F(t)p(s)\,dt\,ds - \int_{a}^{b} \int_{c}^{d}\psi^{4}_{s}(t)k'(s)G(s)w(t)\,ds\,dt. \end{aligned}$$
  Since *ϕ* is convex on $[0,\infty )\times [0,\infty )$, by Lemma 2.3 we conclude that *ϕ* is coordinate convex on $[0,\infty )\times [0, \infty )$, and thus $\psi^{3}_{s}(t)\geq 0$, $\psi^{4}_{s}(t)\geq 0$. Also, *k* and *g* are decreasing, so that $k'(s)\leq 0$ and $g'(t)\leq 0$. Thus it follows that
  3.6 $$ - \int_{c}^{d} \int_{a}^{b} \psi^{3}_{s}(t)g'(t)F(t)p(s)\,dt\,ds \geq 0 $$
  and
  3.7 $$ - \int_{a}^{b} \int_{c}^{d} \psi^{4}_{s}(t)k'(s)G(s)w(t)\,ds\,dt \geq 0. $$
  Combining (3.5), (3.6), and (3.7) yields
  $$\begin{aligned} \int_{a}^{b} \int_{c}^{d} \bigl[\phi \bigl(f(t),h(s)\bigr)-\phi \bigl(g(t),k(s)\bigr) \bigr]w(t)p(s)\,dt\,ds\geq 0, \end{aligned}$$
  which implies the desired inequality (3.1).
(ii) Inequality (3.2) can be proved in the same way as inequality (3.1). Theorem 3.1 is proved.

 □

### **Theorem** 3.2

*Let* *w* *and* *u* *be positive continuous functions on* $[a,b]$ *and* $[c,d]$, *respectively*, *and let* *f*, *g* *and* *k*, *l* *be positive differentiable functions on* $[a,b]$ *and* $[c,d]$, *respectively*. *Suppose that* $\psi :[0,\infty )\times [0,\infty )\rightarrow \mathbb{R}$ *is a convex function*.

(i) *Let* $f/g$ *and* $l/k$ *be decreasing functions on* $[a,b]$ *and* $[c,d]$, *respectively*. *If* *f* *and* *l* *are increasing functions on* $[a,b]$ *and* $[c,d]$, *respectively*, *then*
  3.8 $$\begin{aligned} &\int_{a}^{b} \int_{c}^{d}\psi \biggl(\frac{f(t)}{\int_{a}^{b}f(t)w(t)\,dt}, \frac{l(s)}{ \int_{c}^{d}l(s)u(s)\,ds} \biggr)w(t)u(s)\,dt\,ds \\ &\quad \leq \int_{a}^{b} \int_{c}^{d}\psi \biggl(\frac{g(t)}{\int_{a} ^{b}g(t)w(t)\,dt}, \frac{k(s)}{\int_{c}^{d}k(s)u(s)\,ds} \biggr)w(t)u(s)\,dt\,ds. \end{aligned}$$
  *If* *g* *and* *k* *are decreasing functions on* $[a, b]$ *and* $[c,d]$, *respectively*, *then the reverse inequality of* (3.8) *holds*.
(ii) *Let* $f/g$ *and* $l/k$ *be increasing functions on* $[a,b]$ *and* $[c,d]$, *respectively*. *If* *g* *and* *k* *are increasing functions on* $[a,b]$ *and* $[c,d]$, *respectively*, *then*
  3.9 $$\begin{aligned} &\int_{a}^{b} \int_{c}^{d}\psi \biggl(\frac{g(t)}{\int_{a}^{b}g(t)w(t)\,dt}, \frac{k(s)}{ \int_{c}^{d}k(s)u(s)\,ds} \biggr)w(t)u(s)\,dt\,ds \\ &\quad \leq \int_{a}^{b} \int_{c}^{d}\psi \biggl(\frac{f(t)}{\int_{a} ^{b}f(t)w(t)\,dt}, \frac{l(s)}{\int_{c}^{d}l(s)u(s)\,ds} \biggr)w(t)u(s)\,dt\,ds. \end{aligned}$$

*If* *f* *and* *l* *are decreasing functions on* $[a, b]$ *and* $[c,d]$, *respectively*, *then the reverse inequality of* (3.9) *holds*.

### Proof

(i) Using Lemma 2.4 with substitution $v(t)= g(t)w(t)$ and $h(t)=f(t)/g(t)$ in (2.3), we obtain
  3.10 $$\begin{aligned} &\int_{a}^{b}f(t)w(t)\,dt \int_{a}^{x}g(t)w(t)\,dt \\ &\quad \leq \int_{a} ^{x}f(t)w(t)\,dt \int_{a}^{b}g(t)w(t)\,dt, \quad x\in [a,b]. \end{aligned}$$
  Also, putting $v(s)=k(s)u(s)$ and $h(s)=l(s)/k(s)$ in (2.3) gives
  3.11 $$ \int_{c}^{d}l(s)u(s)\,ds \int_{c}^{y}k(s)u(s)\,ds\leq \int_{c} ^{y}l(s)u(s)\,ds \int_{c}^{d}k(s)u(s)\,ds, \quad y\in [c,d]. $$
  From (3.10) and (3.11) we deduce that
  3.12 $$\begin{aligned}& \int_{a}^{x} \biggl(\frac{g(t)}{\int_{a}^{b}g(t)w(t)\,dt} \biggr)w(t)\,dt \leq \int_{a}^{x} \biggl(\frac{f(t)}{\int_{a}^{b}f(t)w(t)\,dt} \biggr)w(t)\,dt, \quad x\in [a,b], \end{aligned}$$
  3.13 $$\begin{aligned}& \int_{c}^{y} \biggl(\frac{k(s)}{\int_{c}^{d}k(s)u(s)\,ds} \biggr)u(s)\,ds \leq \int_{c}^{x} \biggl(\frac{l(s)}{\int_{c}^{d}l(s)u(s)\,ds} \biggr)u(s)\,ds, \quad y\in [c,d]. \end{aligned}$$
  Additionally, it is easy to observe that
  3.14 $$\begin{aligned}& \int_{a}^{b} \biggl(\frac{g(t)}{\int_{a}^{b}g(t)w(t)\,dt} \biggr)w(t)\,dt= \int_{a}^{b} \biggl(\frac{f(t)}{\int_{a}^{b}f(t)w(t)\,dt} \biggr)w(t)\,dt, \end{aligned}$$
  3.15 $$\begin{aligned}& \int_{c}^{d} \biggl(\frac{k(s)}{\int_{c}^{d}k(s)u(s)\,ds} \biggr)u(s)\,ds= \int_{c}^{d} \biggl(\frac{l(s)}{\int_{c}^{d}l(s)u(s)\,ds} \biggr)u(s)\,ds. \end{aligned}$$
  By relations (3.12), (3.13), (3.14), and (3.15), using inequality (3.2) given in Theorem 3.1, we obtain
  $$\begin{aligned} & \int_{a}^{b} \int_{c}^{d}\psi \biggl(\frac{f(t)}{\int_{a}^{b}f(t) w(t)\,dt}, \frac{l(s)}{\int_{c}^{d}l(s) u(s)\,ds} \biggr) w(t)u(s)\,dt \,ds \\ &\leq \int_{a}^{b} \int_{c}^{d}\psi \biggl(\frac{g(t)}{\int_{a}^{b}g(t) w(t)\,dt}, \frac{k(s)}{\int_{c}^{d}k(s) u(s)\,ds} \biggr) w(t) u(s)\,dt \,ds, \end{aligned}$$
  which is the desired inequality (3.8).
  Using the majorization relations (3.12), (3.13), (3.14), and (3.15) and inequality (3.1), we get the reversed inequality of (3.8).
(ii) If we perform an interchange $f \longmapsto g$ $(g \longmapsto f)$ and $k\longmapsto l$ $(l\longmapsto k)$, then inequality (3.9) follows immediately from (3.8). The reversed inequality of (3.9) can be deduced from the reversed inequality of (3.8) by using the same interchange. This completes the proof of Theorem 3.2.

 □

## Applications to the generalization of Favard’s inequality

As an application of Theorem 3.2, we establish some Favard-type inequalities for functions defined on rectangles, which generalize the results of Theorem 1.4 described in the Introduction.

### Corollary 4.1

*Let* *w* *and* *u* *be positive continuous functions on* $[a,b]$ *and* $[c,d]$, *respectively*, *and let* *f* *and* *l* *be positive differentiable functions on* $[a,b]$ *and* $[c,d]$, *respectively*. *Suppose that* $\psi :[0,\infty )\times [0,\infty )\rightarrow \mathbb{R}$ *is a convex function*.

(i) *Let* $f(t)/(t-a)$ *and* $l(s)/(s-c)$ *be decreasing functions on* $(a,b]$ *and* $(c,d]$, *respectively*. *If* *f* *and* *l* *are increasing functions on* $[a,b]$ *and* $[c,d]$, *respectively*, *then*
  4.1 $$\begin{aligned} &\int_{a}^{b} \int_{c}^{d}\psi \bigl(f(t),l(s) \bigr)w(t)u(s)\,dt\,ds \\ &\quad \leq \int_{a}^{b} \int_{c}^{d}\psi \biggl(\frac{\int_{a}^{b}f(t)w(t)\,dt}{ \int_{a}^{b}(t-a)w(t)\,dt} (t-a), \\ &\quad \quad {} \frac{\int_{c}^{d}l(s)u(s)\,ds}{\int _{c}^{d}(s-c)u(s)\,ds}(s-c) \biggr)w(t)u(s)\,dt\,ds. \end{aligned}$$

(ii) *Let* $f(t)/(b-t)$ *and* $l(s)/(d-s)$ *be increasing function on* $[a,b)$ *and* $[c,d)$, *respectively*. *If* *f* *and* *l* *are decreasing functions on* $[a,b]$ *and* $[c,d]$, *respectively*, *then*
  4.2 $$\begin{aligned} &\int_{a}^{b} \int_{c}^{d}\psi \bigl(f(t),l(s) \bigr)w(t)u(s)\,dt\,ds \\ &\quad \leq\int_{a}^{b} \int_{c}^{d}\psi \biggl(\frac{\int_{a}^{b}f(t)w(t)\,dt}{ \int_{a}^{b}(b-t)w(t)\,dt} (b-t), \\ &\quad \quad {} \frac{\int_{c}^{d}l(s)u(s)\,ds}{\int _{c}^{d}(d-s)u(s)\,ds}(d-s) \biggr)w(t)u(s)\,dt\,ds. \end{aligned}$$

### Proof

Note the simple fact that if $\psi (x,y)$ ($\psi :[0,\infty )\times [0, \infty )\rightarrow \mathbb{R}$) is a convex function, then $\psi (\theta_{1} x,\theta_{2}y)$ ($\theta_{1}$, $\theta_{2}>0$) is also a convex function. Using Theorem 3.2 with substitution

$$ \psi ( x, y)\longmapsto \psi \biggl( \biggl( \int_{a}^{b}f(t)w(t)\,dt \biggr) x, \biggl( \int_{c}^{d}l(s)u(s)\,ds \biggr) y \biggr) $$

in inequality (3.8) and choosing $g(t)=t-a$ and $k(s)=s-c$, we get the required inequality (4.1). Applying the above substitution to the reverse inequality of (3.9) and choosing $g(t)=b-t$ and $k(s)=d-s$, we derive inequality (4.2). □

### Corollary 4.2

*Let* *w* *and* *u* *be positive continuous functions on* $[a,b]$ *and* $[c,d]$, *respectively*, *and let* *f* *and* *l* *be positive differentiable functions on* $[a,b]$ *and* $[c,d]$, *respectively*. *Suppose that* $\psi :[0,\infty )\times [0,\infty )\rightarrow \mathbb{R}$ *is a convex function*.

(i) *Let* $f(t)/(t-a)$ *and* $l(s)/(s-c)$ *be decreasing functions on* $(a,b]$ *and* $(c,d]$, *respectively*. *If* *f* *and* *l* *are increasing functions on* $[a,b]$ *and* $[c,d]$, *respectively*, *then*
  4.3 $$\begin{aligned} &\frac{1}{(b-a)(d-c)}\int_{a}^{b} \int_{c}^{d}\psi \bigl(f(t),l(s) \bigr)w(t)u(s)\,dt\,ds \\ &\quad\leq \int_{0}^{1} \int_{0}^{1}\psi (\xi \widetilde{f}, \eta \widetilde{l}) w \bigl(a(1-\xi )+b\xi \bigr)u \bigl(c(1-\eta )+d \eta \bigr)\,d\xi \,d\eta , \end{aligned}$$
  *where* $\widetilde{f}=(b-a)\int_{a}^{b} f(t)w(t)\,dt / \int_{a}^{b} (t-a)w(t)\,dt$ *and* $\widetilde{l}=(d-c)\int_{c}^{d} l(s)u(s)\,ds / \int_{c}^{d} (s-c)u(s)\,ds$.
(ii) *Let* $f(t)/(b-t)$ *and* $l(s)/(d-s)$ *be increasing functions on* $[a,b)$ *and* $[c,d)$, *respectively*. *If* *f* *and* *l* *are decreasing functions on* $[a,b]$ *and* $[c,d]$, *respectively*, *then*
  4.4 $$\begin{aligned} &\frac{1}{(b-a)(d-c)} \int_{a}^{b} \int_{c}^{d}\psi \bigl(f(t),l(s) \bigr)w(t)u(s)\,dt\,ds \\ &\quad \leq \int_{0}^{1} \int_{0}^{1}\psi (\xi \widetilde{f}, \eta \widetilde{l}) w \bigl(a\xi +b(1-\xi ) \bigr)u \bigl(c\eta +d(1- \eta ) \bigr)\,d\xi \,d\eta , \end{aligned}$$
  *where* $\widetilde{f}=(b-a)\int_{a}^{b} f(t)w(t)\,dt / \int_{a}^{b} (b-t)w(t)\,dt$ *and* $\widetilde{l}=(d-c)\int_{c}^{d} l(s)u(s)\,ds / \int_{c}^{d} (d-s)u(s)\,ds$.

### Proof

Substituting $\xi =\frac{t-a}{b-a}$ and $\eta =\frac{s-c}{d-c}$ into the right-hand sides of (4.1), we get

$$\begin{aligned} &\int_{a}^{b} \int_{c}^{d}\psi \biggl(\frac{\int_{a}^{b}f(t)w(t)\,dt}{\int _{a}^{b}(t-a)w(t)\,dt} (t-a), \frac{\int_{c}^{d}l(s)u(s)\,ds}{\int_{c} ^{d}(s-c)u(s)\,ds}(s-c) \biggr)w(t)l(s)\,dt\,ds \\ &\quad = \int_{0}^{1} \int_{0}^{1}\psi \biggl(\xi \frac{(b-a)\int_{a}^{b}f(t)w(t)\,dt}{ \int_{a}^{b}(t-a)w(t)\,dt}, \\ &\quad \quad {}\eta \frac{(d-c)\int_{c}^{d}l(s)u(s)\,ds}{ \int_{c}^{d}(s-c)u(s)\,ds} \biggr)w \bigl(a(1-\xi )+b\xi \bigr)l \bigl(c(1- \eta )+\,d\eta \bigr)\,d\xi \,d\eta , \end{aligned}$$

which, together with inequality (4.1), leads to the desired inequality (4.3).

Similarly, we can deduce inequality (4.4) by substituting $\xi =\frac{b-t}{b-a}$ and $\eta =\frac{d-s}{d-c}$ into the right-hand sides of (4.2). □

### Corollary 4.3

*Let* *w* *and* *u* *be positive continuous functions on* $[a, b]$ *and* $[c,d]$, *respectively*. *Suppose that* $\psi :[0,\infty )\times [0, \infty )\rightarrow \mathbb{R}$ *is a convex function*.

(i) *If* *f* *and* *l* *are positive increasing concave functions on* $[a,b]$ *and* $[c,d]$, *respectively*, *then*
  4.5 $$\begin{aligned}& \frac{1}{(b-a)(d-c)} \int_{a}^{b} \int_{c}^{d}\psi \bigl(f(t),l(s) \bigr)w(t)u(s)\,dt\,ds \\& \quad \leq \int_{0}^{1} \int_{0}^{1}\psi (\xi \widetilde{f}, \eta \widetilde{l}) w \bigl(a(1-\xi )+b\xi \bigr)u \bigl(c(1-\eta )+d \eta \bigr)\,d\xi \,d\eta , \end{aligned}$$
  *where* $\widetilde{f}=(b-a)\int_{a}^{b} f(t)w(t)\,dt / \int_{a}^{b} (t-a)w(t)\,dt$ *and* $\widetilde{l}=(d-c)\int_{c}^{d} l(s)u(s)\,ds /\int_{c}^{d} (s-c)u(s)\,ds$.
(ii) *If* *f* *and* *l* *are positive decreasing concave functions on* $[a,b]$ *and* $[c,d]$, *respectively*, *then*
  4.6 $$\begin{aligned}& \frac{1}{(b-a)(d-c)} \int_{a}^{b} \int_{c}^{d}\psi \bigl(f(t),l(s) \bigr)w(t)u(s)\,dt\,ds \\& \quad \leq \int_{0}^{1} \int_{0}^{1}\psi (\xi \widetilde{f}, \eta\widetilde{l}) w \bigl(a\xi +b(1-\xi ) \bigr)u \bigl(c\eta +d(1- \eta ) \bigr)\,d\xi \,d\eta , \end{aligned}$$

*where* $\widetilde{f}=(b-a)\int_{a}^{b} f(t)w(t)\,dt / \int_{a}^{b} (b-t)w(t)\,dt$ *and* $\widetilde{l}=(d-c)\int_{c}^{d} l(s)u(s)\,ds / \int_{c}^{d} (d-s)u(s)\,ds$.

### Proof

(i) By the first part of Corollary 4.2, to prove inequality (4.5), it suffices to prove that $f(t)/(t-a)$ and $l(s)/(s-c)$ are decreasing functions on $(a,b]$ and $(c,d]$, respectively.
  Consider the function
  $$ P(t)=\frac{f(t)}{t-a},\quad t\in (a,b]. $$
  Differentiation of $P(t)$ with respect to *t* gives
  $$\begin{aligned} P'(t)&=\frac{f'(t)(t-a)-f(t)}{(t-a)^{2}} \\ & =\frac{f'(t)(t-a)-f(t)+f(a)}{(t-a)^{2}}-\frac{f(a)}{(t-a)^{2}} \\ & =\frac{f'(t)-\frac{f(t)-f(a)}{t-a}}{t-a}-\frac{f(a)}{(t-a)^{2}} \\ & =\frac{f'(t)-f'(\theta )}{t-a}-\frac{f(a)}{(t-a)^{2}} \quad (a< \theta < t). \end{aligned}$$
  It follows that $P'(t)<0$ for $t\in (a,b]$ since *f* is a positive concave function on $[a,b]$. Thus we conclude that $P(t)=f(t)/(t-a)$ is a decreasing function on $(a,b]$. In the same way, we can prove that $l(s)/(s-c)$ is a decreasing function on $(c,d]$. This proves inequality (4.5).
(ii) Similarly, we have
  $$ Q'(t)= \biggl(\frac{f(t)}{b-t} \biggr)'= \frac{f'(t)-f'(\zeta )}{b-t}+ \frac{f(a)}{(b-t)^{2}} \quad (t< \zeta < b). $$
  We deduce that $Q'(t)>0$ for $t\in [a,b)$, since *f* is positive concave function on $[a,b]$. Thus, $Q(t)=f(t)/(b-t)$ is an increasing function on $[a,b)$. In the same way, we can prove that $l(s)/(d-s)$ is an increasing function on $[c,d)$. Therefore inequality (4.6) follows from the second part of Corollary 4.2.

 □

## Final remarks

Obviously, the results of Corollary 4.3 are generalizations of those given in Theorem 1.6 relating to Favard’s inequality. Indeed, if we put $w(t)=1$, $u(s)=1$, $f=\Phi $, and $\psi (x,y)= \Upsilon (x)$ in (4.5) and (4.6), respectively, then we obtain the Favard inequality (1.6). Further, we can deduce the Favard inequality (1.7) by taking $\Upsilon (x)=x^{q}$ ($q>1$). It is worth noting that the majorization inequalities asserted in Theorem 3.1 play a key role in proving Theorem 3.2. Further, with the help of Theorem 3.2, we obtain some significant results in Corollaries 4.1, 4.2, and 4.3.
